# Cross-talk mechanism between endothelial cells and hepatocellular carcinoma cells via growth factors and integrin pathway promotes tumor angiogenesis and cell migration

**DOI:** 10.18632/oncotarget.18632

**Published:** 2017-06-27

**Authors:** Tang Feng, Hongchi Yu, Qing Xia, Yunlong Ma, Hongmei Yin, Yang Shen, Xiaoheng Liu

**Affiliations:** ^1^ Institute of Biomedical Engineering, School of Preclinical and Forensic Medicine, Sichuan University, Chengdu 610041, China; ^2^ West China School of Pharmacy, Sichuan University, Chengdu 610041, China

**Keywords:** vascular endothelial cells, hepatocellular carcinoma cells, cross-talk, tumor angiogenesis, cell migration

## Abstract

Tumor angiogenesis plays a central role in the development and metastasis of hepatocellular carcinoma. Cancer cells secrete angiogenic factors to recruit vascular endothelial cells and sustain tumor vascular networks, which facilitate the migration and invasion of cancer cells. Therefore, the cross-talk between vascular endothelial cells and cancer cells is vitally necessary, however, little is known about the cross-talk mechanism of these cells interaction. In the present study, the proliferation, migration, invasion and tube formation of vascular endothelial EA.hy926 cells and hepatocellular carcinoma HepG2 cells were studied by exchanging their culture medium. The time-dependent differences of integrins induced signaling pathway associated with cell migration were investigated. Our results showed that HepG2 cells markedly enhanced the proliferation and migration ability as well as the tube formation of EA.hy926 cells by releasing growth factors. Also, the EA.hy926 cells promoted the proliferation, migration and invasion ability of HepG2 cells. The further analysis demonstrated that the integrins-FAK-Rho GTPases signaling events in both of two cells was activated under conditioned medium, and the signaling molecules in two cell lines showed a different time-dependent expression within 1h. These findings reveal the cross-talk mechanism between the endothelial cells and hepatocellular carcinoma cells, which were expected to find out new ideas for the prevention and treatment of hepatocellular carcinoma.

## INTRODUCTION

As an essential ingredient of cancer malignancy, tumor microenvironment certainly plays a determining role in development and progression of tumors. In the tumor microenvironment, tumor angiogenesis is a multistep process that involves integration of several signaling contributing to the migration of endothelial cells and the invasion of cancer cells [1]. The cross-talk between tumor and endothelial cells alters their properties and facilitates the invasive behavior of tumor cells. It is well known that cancer cells secrete angiogenic factors to recruit endothelial cells and sustain tumor vascular networks [2]. The migration of vascular endothelial cells (VECs) is a fundamental process contributing to tumor angiogenesis. The tumor vascularization is induced by pro-angiogenic factors to facilitate the migration and proliferation of VECs [3]. During tumor angiogenesis, extracellular matrix (ECM) is deposited to form basement membrane to surround blood vessels. Cancer cells release matrix metalloproteinases (MMPs) to degrade endothelial basement membrane and ECM components in tumor blood vessel, which facilitate cancer cells migrate and invade into the circulation, and finally form new metastatic tumors in surrounding tissues [4–6]. Therefore, the cross-talk of tumor cells and vascular endothelial cells participates in the whole process of tumor angiogenesis. However, little is known about the effect of endothelial cell-secreted factors on the behaviors and functions of tumor cells.

Globally, hepatocellular carcinoma (HCC) is ranking fifth the most common cancer in men and the seventh in women [7], and due to the rapid progression and high lethality of HCC, it is important to identify novel targets and develop new therapeutic strategies in order to improve the survival rate of HCC patients. HCC is a complex and heterogeneous tumor most commonly associated with underlying chronic liver disease. HCC is a hyper vascular tumor, and the vascular endothelial growth factor (VEGF) and angiopoietins are important endothelium-specific growth factor families in HCC [8]. Tumor microenvironment comprises numerous signaling events that influence the angiogenic response. Among angiogenic factors, VEGF is one of the key factors inducing angiogenesis, which is upregulated in most human tumors [9, 10]. Previous studies manifested that VEGF is the most important substance for promoting angiogenesis. VEGF binding to the receptors (Flt-1, Flk-1) initiates the tyrosine kinase signaling pathway, alters the permeability of vessels, and promotes the migration and proliferation of vascular endothelial cells, eventually forms new mature blood vessels. Also, epidermal growth factor (EGF) is a crucial growth factor. By binding to its receptor (EGFR) [11], activating Ras and MAPK signaling pathways, the transcription factor c-fos phosphorylation, produces AP-1 and Elk-1, and also promotes cell proliferation and differentiation. Not only inducing tumor angiogenesis, cross-talk between integrin and growth factor receptors is also an important signaling mechanism to provide specificity during cell growth, migration, and invasion [12].

Integrins are transmembrane receptors that bind to ECM through extracellular domains of α subunits on cell membranes [13, 14]. Integrins often do not work in isolation, but in combination with specific receptors, like EGF and VEGF, to form complexes mediating cell function [15]. Around activated integrin clusters, cytoskeletal proteins such as Talin, Paxillin, Vinculin etc., as the ligands of β integrin cytoplasmic tails assemble together. At the regions of cell–substratum contacts, they form focal adhesion (FA) plaques and provide enough adhesive sites to support stable cell attachment. FA plaque disassembly drives the migration cycle through activating Rho-family GTPases including RhoA, Rac1 and Cdc42. This results in directly local actin assembly by regulating stress fibers, lamellipodia or filopodia. In addition, the integrin-binding Talin and Paxillin recruit focal adhesion kinase (FAK) via C-terminal focal-adhesion targeting domain and phosphorylated FAK at initial Y397 tyrosine site, which leads to a cascade of activation of other downstream signals. Consequently, intercellular FA formation, activation of Rho-family GTPases, and associated with cellular FAK signaling events likely participate in determining cell adhesion/ migration behavior as well as other biological events.

The activation of the proteins can occur in a very short time with certain physical and chemical stimulus [16]. Within 1 min, changes of the expression of VEGF could be detectable [17], and integrins were activated at a transient duration by external stimulus [18]. In this study, the proliferation, migration, invasion and tube formation of vascular endothelial EA.hy926 cells and hepatocellular carcinoma HepG2 cells were studied by exchanging their culture medium. The time-dependent differences of integrins induced signaling pathway associated with cell migration were further investigated. Examination of the interaction between endothelial cells and hepatocellular carcinoma cells could help us to better understand the mechanism of tumor angiogenesis, which is expected to find new therapeutic strategies.

## RESULTS

### The cross-talk between endothelial cells and hepatocellular carcinoma cells enhanced the proliferation and migration ability

**Figure 1:**
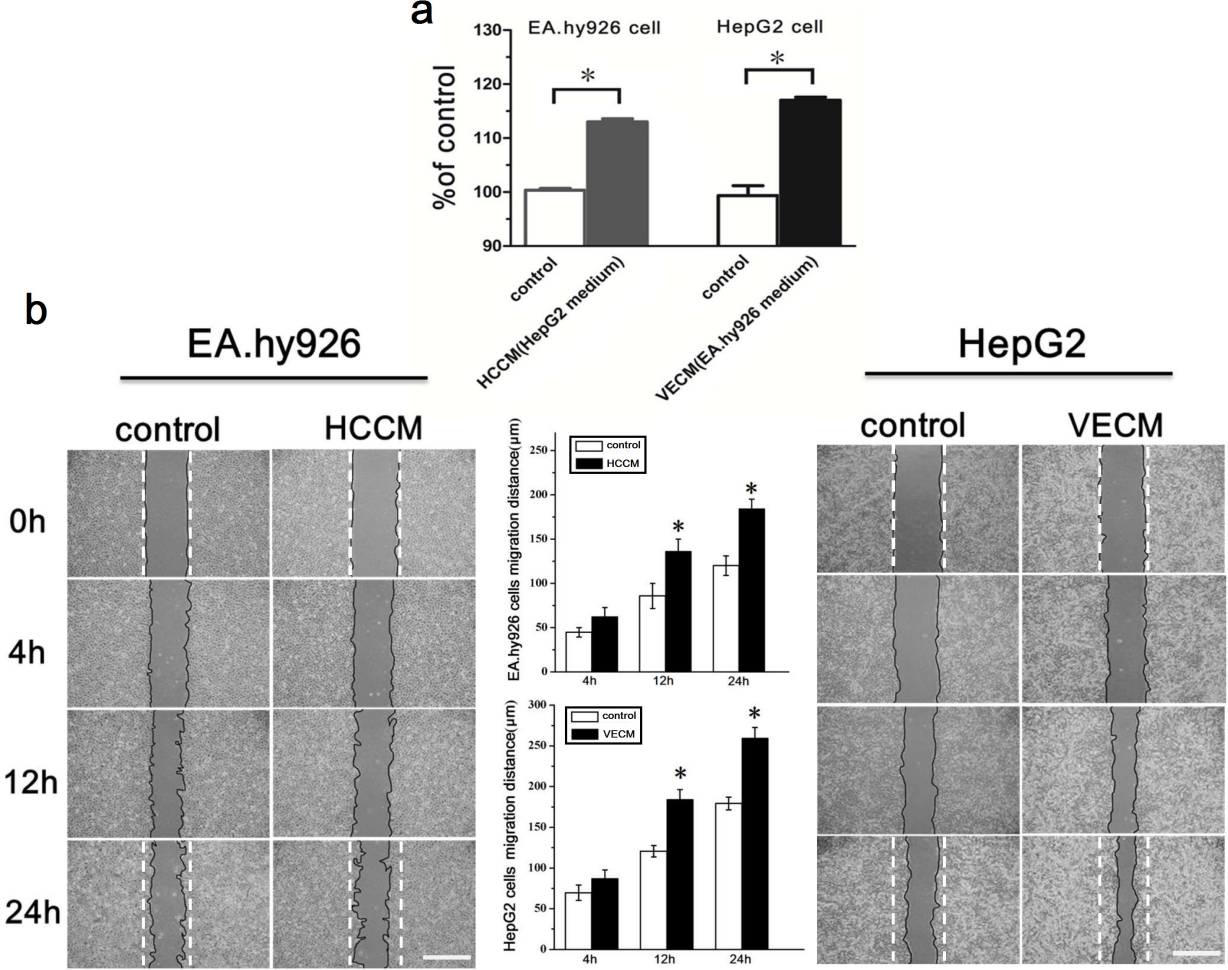
The proliferation and migration of EA.hy926 cells and HepG2 cells were enhanced under the conditioned medium **(a)** The effect of hepatocellular carcinoma HepG2 cell culture medium (HCCM) on the proliferation of EA.hy926 cells, as well as the effect of vascular endothelial EA.hy926 cells medium (VECM) on the proliferation of HepG2 cells. **(b)** The typical scratch wound images of EA.hy926 and HepG2 cells migration and the statistical results of average cell migration distance based on images, scale bar=500 μm. *, *P*<0.05 denotes statistically significant difference compare with other groups.

The proliferation of endothelial EA.hy926 cells and hepatoma carcinoma HepG2 cells under the conditioned medium (by exchanging their respective culture medium) were examined using MTT assays. It could be found that, in EA.hy926 cells, the O.D. value of the HCCM (HepG2 cultured medium) was significantly higher than that of the control group (0.254±0.038 vs. 0.229±0.028, *P*<0.05). As to HepG2 cells, VECM (EA.hy926 cultured medium) also markedly increased the O.D. value (0.578±0.026) compared to control group (0.486±0.024). The ratio of O.D. value were normalized (% of control) and shown in Figure 1a. Then we explored the ability of cell migration under the conditioned medium using scratch wound assays. As seen in Figure 1b, the migration distances of two cell lines exposed to exchanging culture medium were significantly longer than that of the control group at 12h. These results showed that the proliferation and migration of HepG2 cells and EA.hy926 cells could be markedly improved under the condition of tumor microenvironment formed by the interaction of vascular endothelial cells and hepatoma cells.

### HepG2 cells enhanced tube formation of endothelial cells

**Figure 2:**
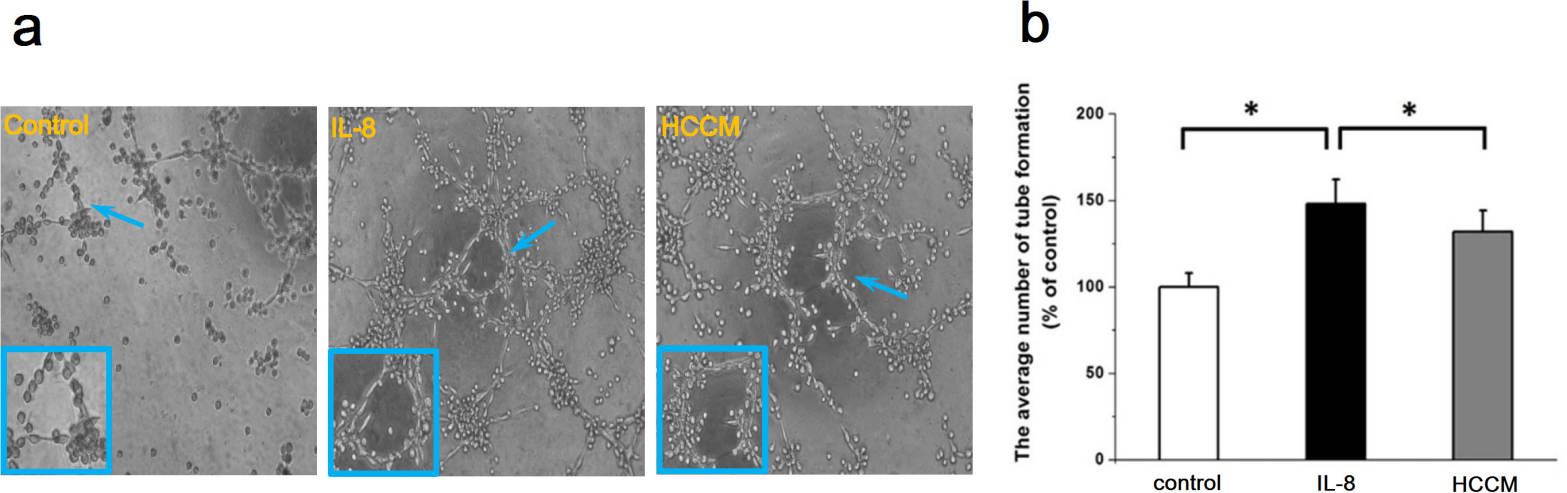
HCCM enhanced the tube formation of EA.hy926 cells **(a)** The typical images showed the tube formation of EA.hy926 cells stimulated by IL-8 and HCCM, scale bar=100 μm. **(b)** The statistical result of average number of tubes. *, *P*<0.05 denotes statistically significant difference compare with other groups.

To study angiogenesis of endothelial cells, the tube formation of endothelial EA.hy926 cells cultured with HCCM was evaluated. As shown in Figure 2a, it could be found that the endothelial cells showed lumen-like distribution in all groups. This lumen-like structure could not be found with addition of Wortmannin (PI3K inhibitor) and Y15 (FAK inhibitor) (Supplementary Figure 1). Compared with control, both of HCCM and the positive interleukin-8 (IL-8) group exhibited clear tube-like formation and longer diameter of the lumen (indicated by enlarged images in Figure 2a). The statistical analysis showed that the numbers of small tube-like structure in HCCM group were significantly more than that in control, but less than that in positive control IL-8 group (Figure 2b). This result provided strong evidences that hepatoma cells can activate the VECs and promote angiogenesis by releasing a lot of growth factors, suggesting that the interaction of hepatoma cells and endothelial cells in tumor microenvironment can facilitate tumor metastasis.

### HCCM increased the VEGF and EGF expressions of EA.hy926 cells

**Figure 3:**
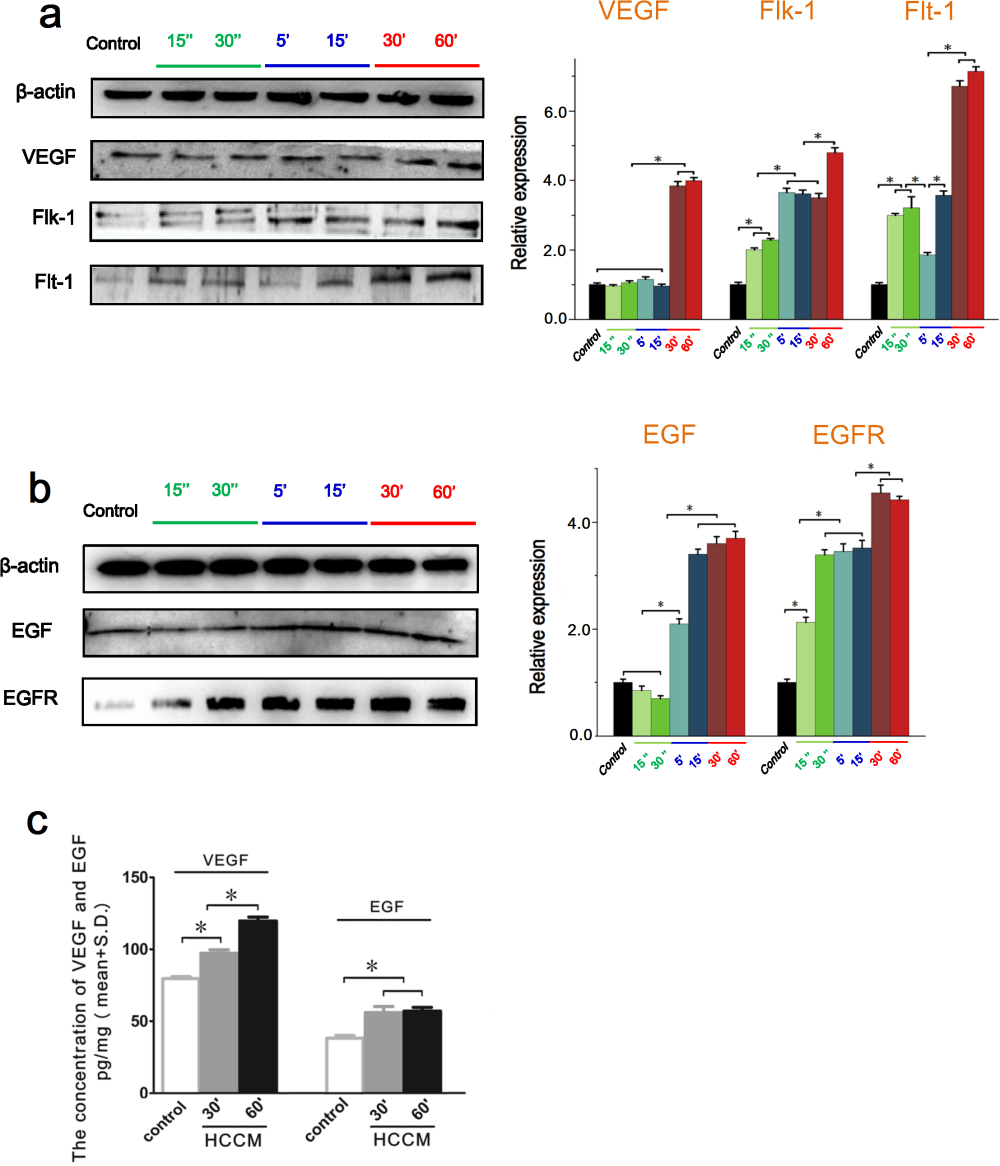
The intracellular expression and the extracellular content of VEGF/EGF in endothelial EA.hy926 cells with HCCM **(a)** The expression of VEGF, Flt-1 and Flk-1 in EA.hy926 cells. Their expression levels were quantified by image analysis of the Western blot bands. The expression of β-actin in each group was used as intrinsic controls, and relative expressions of VEGF, Flt-1 and Flk-1 were calculated. **(b)** The expression of EGF and EGFR in EA.hy926 cells. The expression levels were quantified by image analysis of the Western blot bands. The expression of β-actin in each group was used as intrinsic controls, and relative expressions of EGFand EGFR were calculated. **(c)** The concentration of the VEGF and EGF secreted by EA.hy926 cells with HCCM. The initial concentrations of VEGF and EGF in HCCM are defined as controls. Values represent the Mean±S.D from three independent experiments. *, *P*<0.05 denotes statistically significant difference compare with other groups.

As is known, VEGF and EGF play a central role in the process of tumor angiogenesis. Previous results of MTT and tube formation assay showed that HepG2 cells culture medium promoted the proliferation and angiogenesis of endothelial EA.hy926 cells. Consequently, the intracellular level and extracellular content of VEGF/EGF in EA.hy926 cells were examined using Western blot and ELISA assay. As shown in Figure 3a, the HCCM significantly upregulated the intracellular VEGF expression in EA.hy926 cells at 30 min, and maintained a relative higher level until 60 min. As one of VEGF receptors, intracellular Flk-1 of EA.hy926 cells expression increased with addition of HCCM in a time-dependent tendency. It was significantly upregulated at initial 15 sec compared to control, reaching to maximum at 60 min. Similarly, another VEGF receptor Flt-1 was also increased at initial 15 sec, and then gradually increased within 1h.

Also, the HCCM enhanced intracellular EGF and EGFR level of EA.hy926 in time-dependent way. The expression of EGF was significantly increased after 5 min compared with control (*P*<0.05), and maintained a relative higher level from 15 min to 1h. The receptor of EGF, EGFR was increased at initial 15 sec, and gradually upregulated within 1h. We further examined the extracellular VEGF/EGF in medium released by EA.hy926 using ELISA assay (Figure 3b). It could be found that the extracellular concentrations of both VEGF/EGF were significantly higher than that of the control (Figure 3c). These results suggested HCCM up-regulated intracellular and extracellular VEGF/EGF level in endothelial cells, which result in activating endothelial cells to enhance their proliferation, migration and tube formation.

### Endothelial EA.hy 926 cells enhanced HepG2 cells invasion

**Figure 4:**
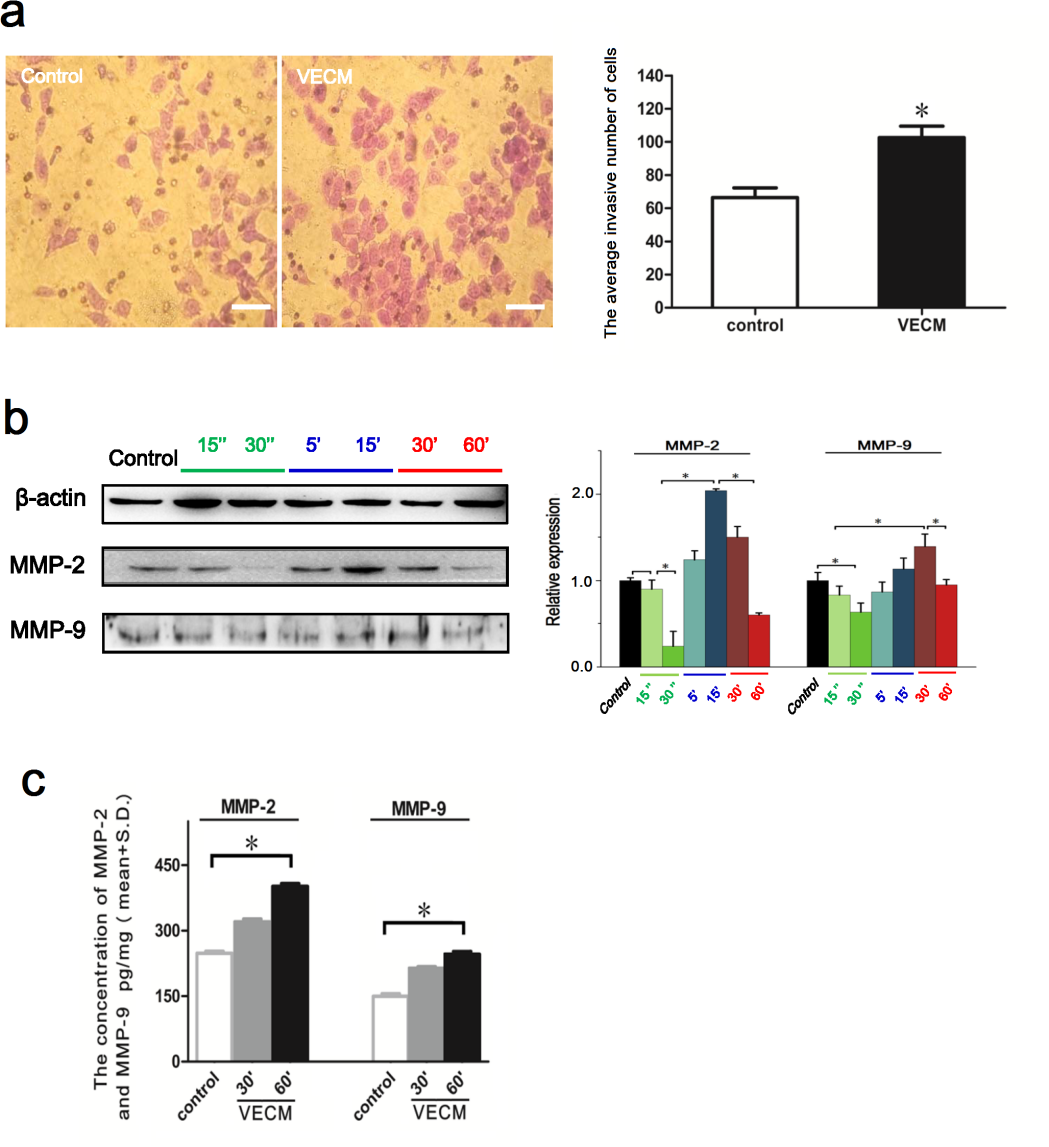
The effects of EA.hy926 conditioned medium on the invasive ability of HepG2 cells **(a)** Typical optical images of HepG2 cells which crossed through the pores of Transwell chamber and stained with crystal violet, illustrating invasive cells stimulated by VECM. Scale bar=30μm. The numbers of invasive cells from the images were quantitatively analyzed. *, *P*<0.05 denotes statistically significant difference compare with control. **(b)** The expressions of MMP-2 and MMP-9 in HepG2 cells. Their expression levels were quantified based on image analysis of the Western blot bands. *, *P*<0.05 denotes statistically significant difference compare with other groups. **(c)** The concentration of the MMP-2 and MMP-9 secreted by HepG2 cells with VECM. The initial concentrations of MMP-2 and MMP-9 in HCCM are defined as controls. Values represent the Mean±S.D from three independent experiments.

We established Transwell model to detect the effect of EA.hy926 cells culture medium on the invasion ability of HepG2 cells. After 24h stimulation, it could be found that endothelial cells culture medium induced efficient HepG2 cells invasion through the filter of Transwell chamber. There was a significant difference of invasive HepG2 cells between the VECM group and control (Figure 4a), suggesting that the invasion ability of hepatoma cells could be markedly enhanced under the influence of secretions in VECM.

MMP-2 and MMP-9 can specifically degrade the type IV collagen which is the dominant component in ECM, facilitating tumor cell migration and metastasis. Accordingly, intracellular expression of MMP-2 and MMP-9 in HepG2 cells and their concentrations in VECM were investigated. Our results showed that with the prolongation of the time in the stimulation of the exchanging medium, the intracellular expressions of MMP-2 and MMP-9 were significantly upregulated at 15 min and 30 min, respectively (Figure 4b). Also, the ELISA results indicated that the extracellular concentrations of MMP-2 and MMP-9 significantly higher than that of the control at 60 min (Figure 4c). These results were consistent with the results of Transwell invasion assay, indicating that VECM enhanced HepG2 invasion by upregulating intracellular and extracellular MMPs levels.

### EA.hy926 cells increased the intracellular expression and the extracellular content of VEGF, EGF of HepG2 cells

**Figure 5:**
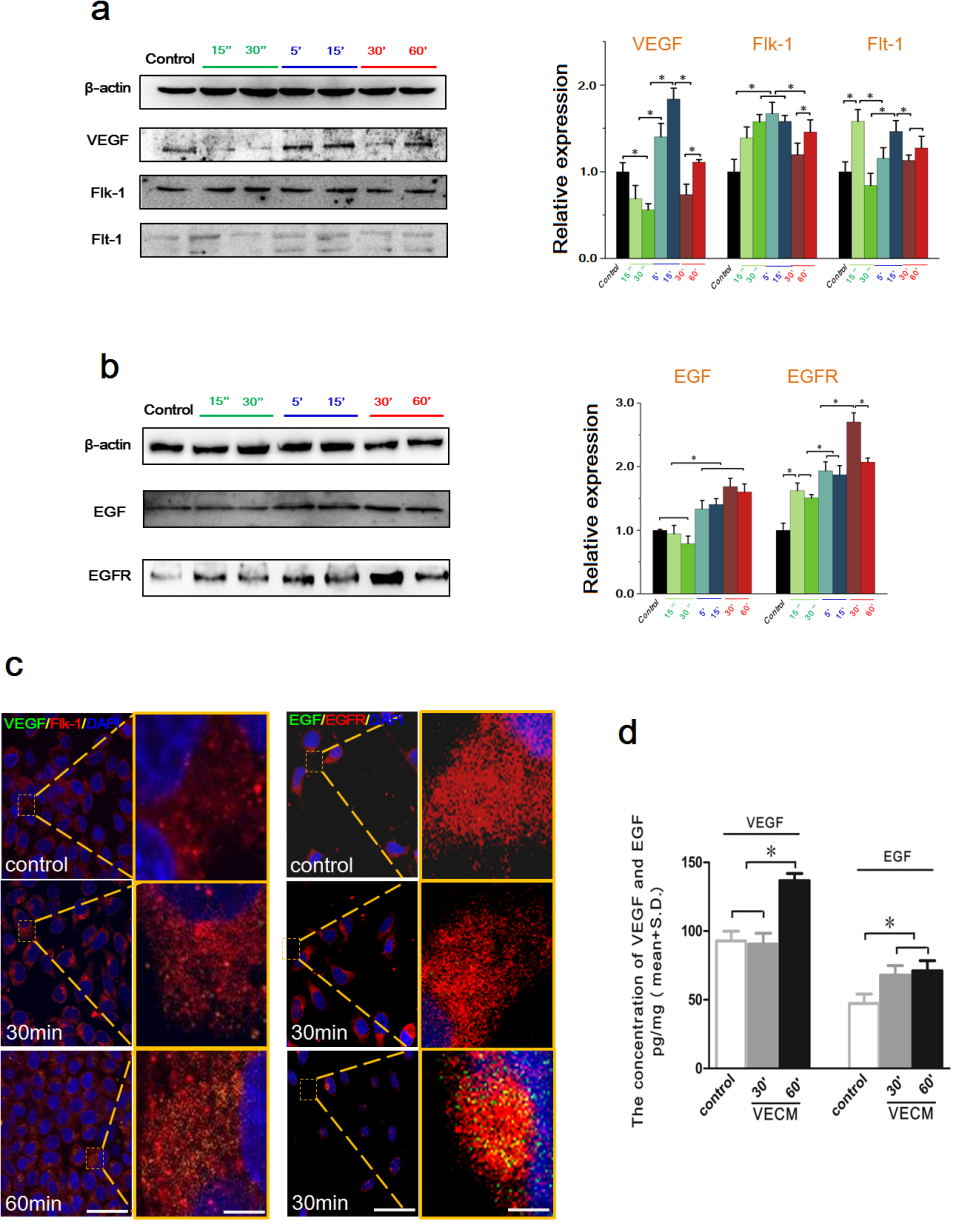
The intracellular expression and the extracellular content of VEGF/EGF in HepG2 cells with VECM **(a)** The expression of VEGF, Flt-1 and Flk-1 in HepG2 cells. **(b)** The expression of EGF and EGFR in HepG2 cells. The expression levels were quantified by image analysis of the Western blot bands. The expression of β-actin in each group was used as intrinsic controls, and relative expressions were calculated. **(c)** The ligand binding of VEGF (green) /Flk-1 (red) and EGF (green)/EGFR (red). The enlarged images of designated regions showed the co-location of ligands (VEGF and EGF) and their receptors (Flk-1 and EGFR), respectively. Scale bar=500 μm. **(d)** The concentration of the VEGF and EGF secreted by HepG2 cells with VECM. The initial concentrations of VEGF and EGF in VECM are defined as controls. Values represent the Mean±S.D from three independent experiments. *, *P*<0.05 denotes statistically significant difference compare with other groups.

We also examined the intracellular expression and the extracellular content of VEGF, EGF and their receptors in HepG2 cells under the stimulation of VECM. Our results indicated that the intracellular expression of VEGF was sharply decreased at initial 15 and 30 sec, and significantly up-regulated at 5 min. It was equal to the level of control at 1h eventually. Both Flk-1 and Flt-1 were increased from initial 15 sec to 15 min, interestingly, they subsequently down-regulated from 30 min (Figure 5a).

Under the stimulation of VECM, the intracellular expression of EGF in HepG2 cells was significantly stronger than the control from 5 min, and maintained a relative higher level until 1h. Additionally, the EGFR expression was gradually upregulated from 15 sec (*P*<0.05), and showed the highest level at 30 min (Figure 5b).

Furthermore, the binding of VEGF/EGF (green fluorescence) and their respective receptors (red fluorescence) were observed using double immunofluorescence staining. The results revealed that the co-location of VEGF/EGF and their respective receptors (yellow fluorescence, enlarged images shown as yellow square frames in Figure 5c) dependently distributed on the cytomembrane (without permeabilization by Triton X-100). Compared with control, the enlarged images of designated regions showed that yellow fluorescence became stronger from 30 min to 60 min, indicating that the ligand binds of VEGF and EGF were gradually increased (Figure 5c). As is shown in Figure 5d, the result of ELISA assay demonstrated that the extracellular concentration of VEGF and EGF were significantly increased at 60 min, however, there was no significant difference of VEGF concentration between VECM stimulation for 30 min and control (the initial concentrations of VEGF in VECM).

### The morphology of cytoskeletons —— F-actin staining

**Figure 6:**
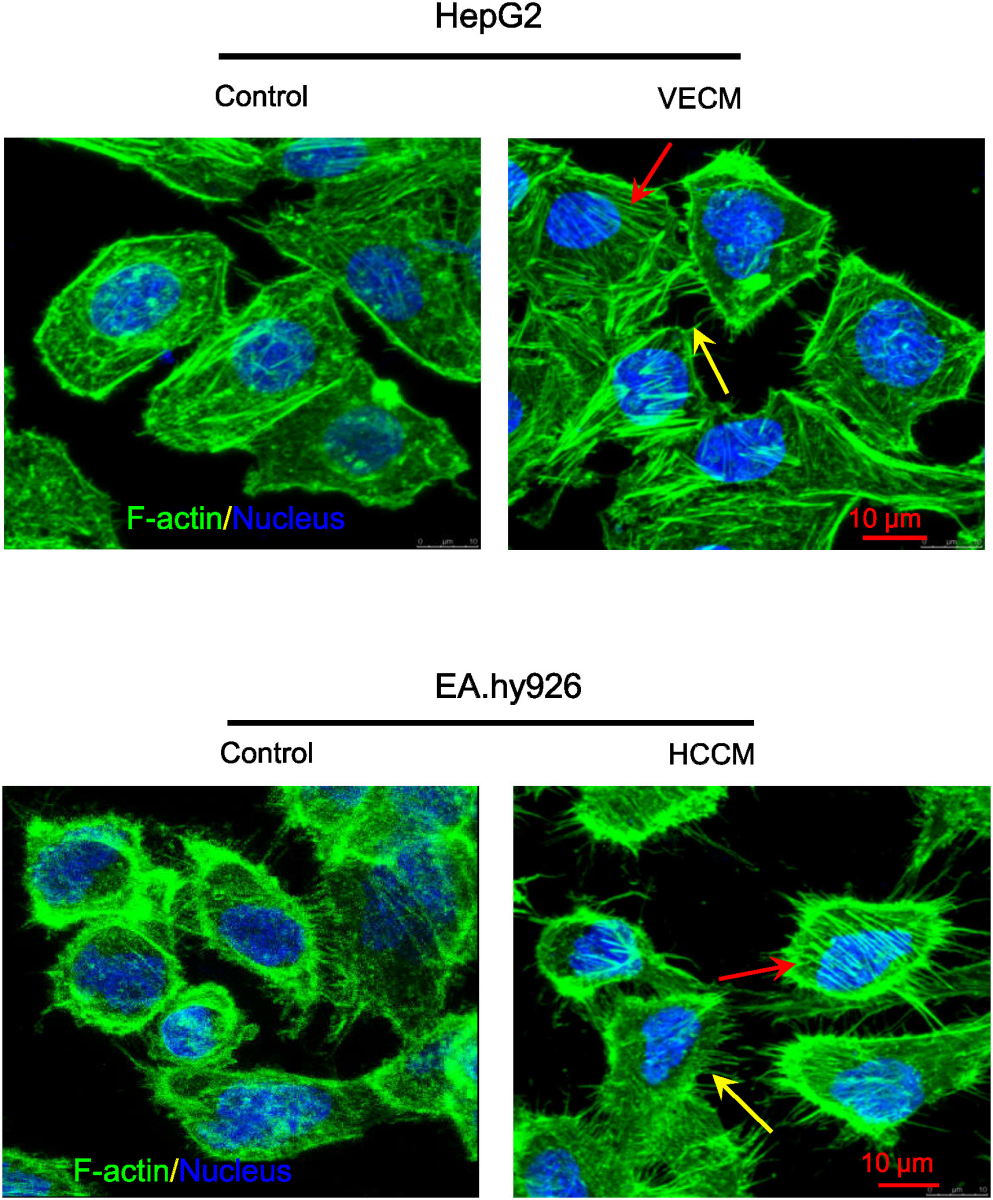
The HCCM and VECM induced F-actin distribution and arrangement in EA.hy926 cells and HepG2 cells, respectively The red and yellow arrows in figures showed the fiber structure in the cell body and filopodia/lamellipodia at the edge of cell protrusions, respective. green: F-actin; blue: nucleus, scale bar = 10 μm.

The arrangements of cytoskeleton F-actin in EA.hy926 cells and HepG2 cells under the conditioned medium are shown in Figure 6. It is clear that both of these two kind cells were obviously activated with the exchanging medium after 1h. Compared with the control, both of these two kinds cells showed abundant F-actin in protrusions (the filopodia/lamellipodia at the edge of cell protrusions, indicated by yellow arrows), and the cells displayed larger coverage, longer filopodia and more bundles of filaments (the fiber structure were well organized, indicated by red arrows), suggesting that those of which were in favor of directing cell locomotion. By contrast, the control showed less and shorter pseudopodia at the leading edge of lamellipodia. This result consists with our previous results of cell migration and invasion, suggesting that tumor microenvironment can promote the migration of these two kinds of cells.

### HCCM activated integrin-FAK-Rho GTPases signals in EA.hy926 cells

A well-known fact is that the functional activities of growth factors like VEGF and EGF are dynamically and reciprocally controlled by integrin [19]. Integrin-activated downstream FAK has been implicated in tumor cell migration, proliferation and metastasis [20]. We previously demonstrated that the adhesion and migration of vascular endothelial cells and human hepatocellular carcinoma cells are both depended on FAK signals. To recognize the molecular mechanism of HCCM-inducing EA.hy926 cell migration, the integrin-FAK-Rho GTPases signals in EA.hy926 cells were investigated accordingly.

**Figure 7:**
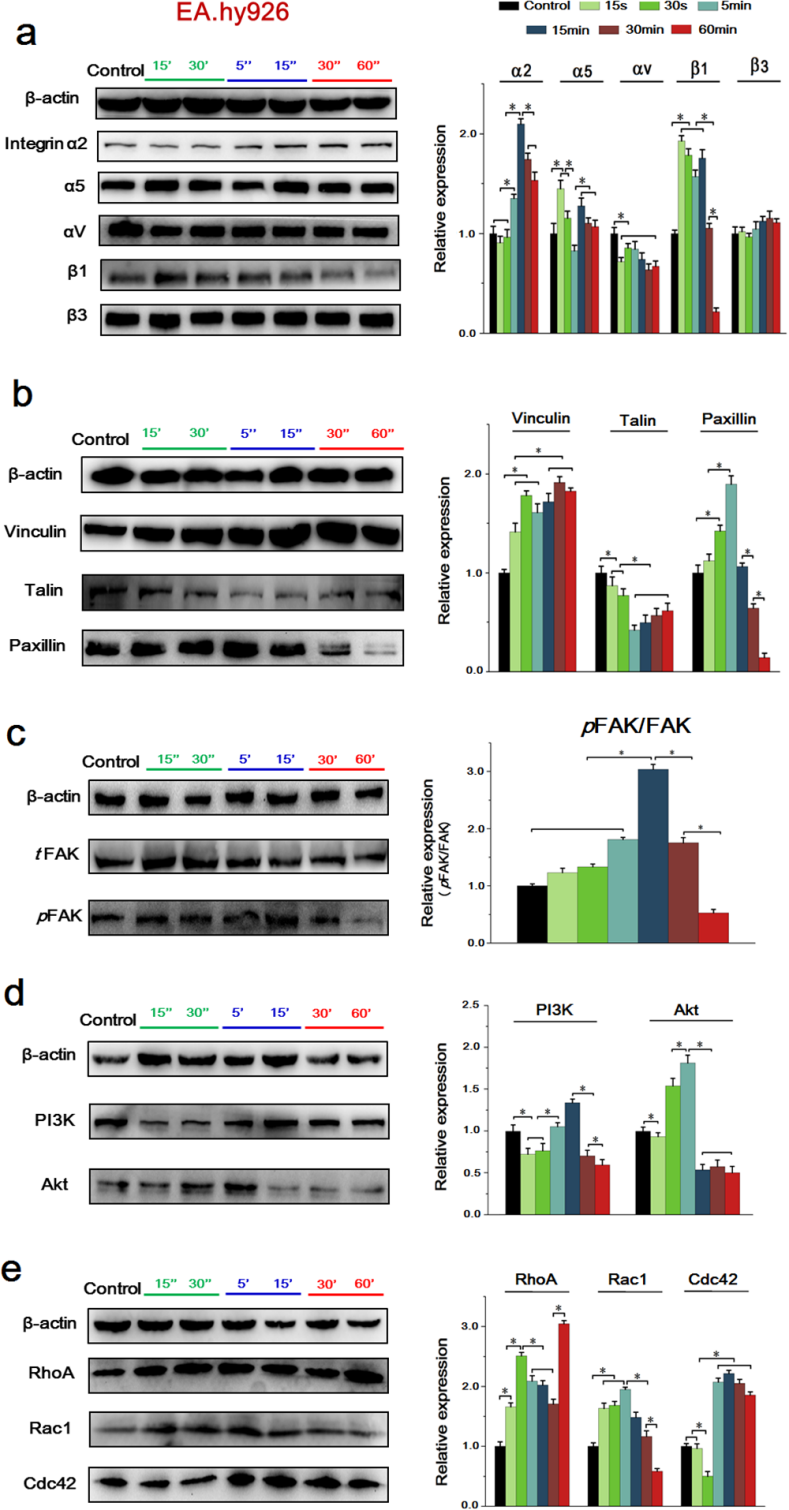
The expressions of key proteins and binding condition in Integrins-FAK-Rho GTPases signaling pathway of EA.hy926 cells **(a)** The expression of integrins, the expression levels were quantified by image analysis of the Western blot bands. The expression of β-actin in each group was used as intrinsic controls, and relative expressions of all of the proteins were calculated. **(b)** The expression of FAs, include Vinculin, Talin and Paxillin. The expression levels were quantified by image analysis of the Western blot bands. **(c)** The expression of total FAK and phosphorylated FAK, their expression levels were quantified based on image analysis of the Western blot bands. **(d)** The expression of PI3K and Akt, the expression levels were quantified based on image analysis of the Western blot bands. **(e)** The expression of Rho GTPases including RhoA, Rac1 and cdc42, as well as the expression levels were quantified by image analysis of the Western blot bands. Values represent the Mean±S.D from three independent experiments. *, *P*<0.05 denotes statistically significant difference compare with other groups.

As shown in Figure 7a, HCCM significantly upregulated the expression of integrin subunit β1 at initial 15 sec, but downregulated it from 30 min. HCCM also increased α5 and α2 integrin at 15 sec and 5 min, respectively. However, it slightly downregulated αV level at 15 sec, and showed no effect on the expression of β3.

As to FA components, Talin, Vinculin and Paxillin play important roles in promoting and stabilizing initial integrin clustering by binding β integrin cytoplasmic tails [21]. Our results showed that, HCCM induced a remarkable increase of Vinculin at initial 15 sec, reaching highest level at 30 min. It also upregulated Paxillin expression from 30 sec, however, Paxillin was sharply decreased from 30 min. As to Talin, it was significantly decreased at first 15 sec, and kept a relative low level until 60 min (Figure 7b).

Also, HCCM activated both of total FAK (*t*FAK) and phosphorylated FAK (*p*FAK) at initial and showed significant difference compared with static control. The ratio of *p*FAK/*t*FAK reached peak at 15 min. Similar to the expression of β1 and Paxillin, FAK level was gradually decreased from 30 min (Figure 7c). By contrast with FAK, HCCM downregulated PI3K at initial 15 sec but upregulated it from 15 min. However, as downstream of PI3K, AKT showed a significant higher level from 30 sec, but was remarkably decreased at 15 min (Figure 7d).

The Rho GTPases, including RhoA, Rac1 and Cdc42, have been shown to regulate many aspects of intracellular actin dynamics, result in directly cell migration eventually [22]. In agreement with upstream molecules, the expressions of RhoA, Rac1 and Cdc42 were enhanced with addition of HCCM. There were significantly differences of RhoA and Rac1 level between 15 sec and static control, while the expression of Cdc42 at 5 min was higher than that at 30 sec. Similar to expression tendency of integrin β1, Paxillin and (*p*) FAK, the Rac1 were sharply decreased from 30 min (Figure 7e).

These results suggested HCCM induced the activation of Rho-GTPases in a time-dependent way, that is to say, activations of RhoA and Rac1 were prior to Cdc42 to mediate endothelial EA.hy 926 cell migration.

### The integrin-FAK-Rho GTPases signaling pathway was activated under the conditioned medium in HepG2 cells

We also investigated the time-dependent difference of integrin-induced signaling cascade in HepG2 cell with the EA.hy926 cell culture medium.

**Figure 8:**
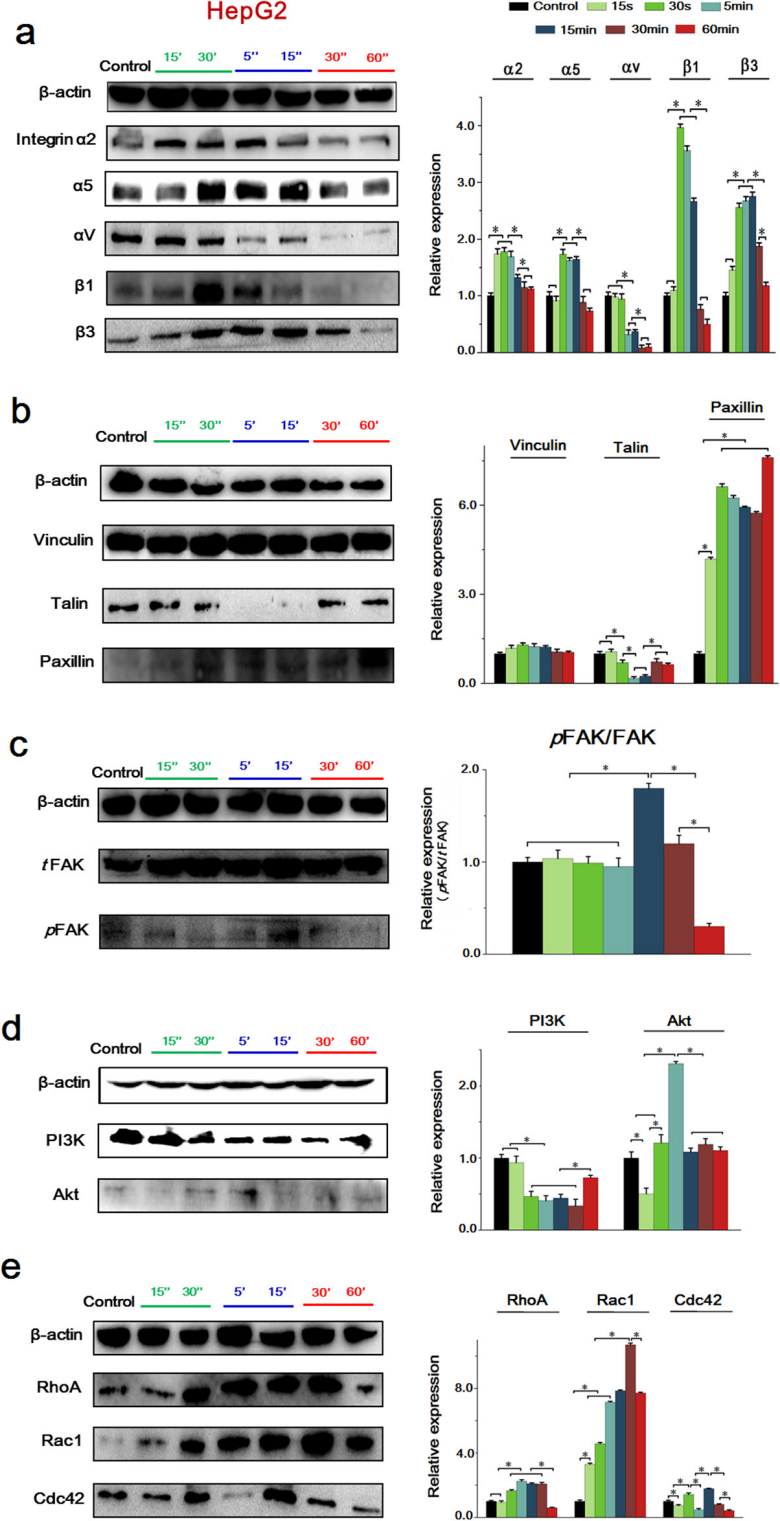
The expressions of key proteins and binding condition in Integrins-FAK-Rho GTPases signaling pathway of HepG2 cells **(a)** The expression of integrins, their expression levels were quantified by image analysis of the Western blot bands. The expression of β-actin in each group was used as intrinsic controls, and relative expressions of all of the proteins were calculated. **(b)** The expression of FAs, include Vinculin, Talin and Paxillin. The expression levels were quantified by image analysis of the Western blot bands. **(c)** The expression of total FAK and phosphorylated FAK, their expression levels were quantified based on image analysis of the Western blot bands. **(d)** The expression of PI3K and Akt, the expression levels were quantified based on image analysis of the Western blot bands. **(e)** The expression of Rho GTPases including RhoA, Rac1 and cdc42, as well as their expression levels were quantified by image analysis of the Western blot bands. Values represent the Mean±S.D from three independent experiments. *, *P*<0.05 denotes statistically significant difference compare with other groups.

As is shown in Figure 8a, the expression of α2 showed a significant increase at initial 15 sec, as well as α5, β1 and β3 were markedly enhanced at 30 sec. Additionally, VECM significantly downregulated αV, which is similar to EA.hy926 cells. Notably, the expression of all these integrin subunits in HepG2 cells was reduced from 30 min, and maintained a low expression until 60 min.

Consistent with EA.hy926 cells, VECM downregulated Talin sharply at 5 min and upregulated Paxillin from 15 sec, maintaining a relative high level as well, while it showed no effect on the expression of Vinculin (Figure 8b). Also, VECM activated *t*FAK and *p*FAK expression and the *p*FAK/*t*FAK showed the maximum at 15 min, but were gradually decreased from 30 min (Figure 8c). The expression of PI3K was inhibited at first 15 sec, while AKT showed a significant higher level from 30 sec, but was remarkably decreased at 15 min (Figure 8d). As to Rho GTPases, HCCM could significantly upregulated Rac1 at 15 sec, RhoA and Cdc42 at 30 sec, respectively. However, all of them showed a sharp decrease at 60 min (Figure 8e).

In general, we can conclude that exchanging culture mediums enhanced cell migration depending on integrin-FAK-Rho GTPases signaling events in both of EA.hy926 cells and HepG2 cells, nevertheless, the molecular expression in these two cells showed a time-dependent difference.

## DISCUSSION

Angiogenesis is generally associated with tumor growth, development and metastasis. As is well-known, the tumor comprises a set of hepatocellular carcinoma cells, a network of dilated and heterogeneous blood vessels (hyper- and hypo- vascularization) of tortuous shape and lymphatic vessels, as well as host cells and extracellular matrix, which contributes to the development of the tumor environment [23]. Tumor microenvironment plays an important role in conducing to the survival and development of HCC. As the critical parts of tumor microenvironment of HCC, the interaction of tumor cells and endothelial cells participates in the whole process of tumor angiogenesis.

Direct co-culture is a method to examine the interaction of two different cells through direct cell-cell contact. Using this model, Ding *et al* [24] studied the interaction between liver cancer cells and human umbilical vein endothelial cells co-cultured in one dish. Indirect co-culture system was built with a Transwell chamber which can be inserted into 6-well plates. In Luo’s study, the mesenchymal stem cells and tenocytes were seeded on culture polystyrene plates and Transwell chamber, respectively [25]. Different from above, exchanging the culture medium is a simple and convenient method to study the cross-talk between different cells. Kristy A. Warner *et al* [26] used a co-culture method to examine the invasiveness of Oral squamous cell carcinoma-3 cells and Kaposi’s sarcoma cells after co-cultured with pools of human dermal microvascular endothelial cells. In this study, we exchanged the culture medium of the vascular endothelial EA.hy926 cells and the hepatocellular carcinoma HepG2 cells. Using this interaction model, therefore, we could explore important events occurred during the tumor development like tumor angiogenesis, invasion and metastasis, which involved cell proliferation and migration, and degradation of extracellular matrix.

Cell migration is necessary for tumor invasion and metastasis. The crucial procedure for most types of cell migration is the formation of the membrane protrusions such as filopodia, lamellipodia together with invadopodia at the leading edge, which are accomplished by filamentous actin dynamic remodeling the basement membrane [27]. In this study, we found that, the migration distances of EA.hy926 cells and HepG2 cells exposed to exchanging culture medium were significantly longer than that of the control group at 12h and 24h, which means that the migration of these two cells could be notably enhanced under the condition of tumor microenvironment formed by the interaction of vascular endothelial cells and hepatoma cells. Additionally, we found that the actin cytoskeleton was rearranged with the stimulation of the exchanging medium. With increased duration of exposure to the exchanging medium, more lamellipodia and flopodia could be found at the edge of cell protrusions, and well-organized F-actin was abundantly accumulated in cell body, indicating that cells could achieve a migratory and invasive phenotype for crossing tissue barriers and thereby reaching blood and lymphatic vessels.

Moreover, metastases represent the end products of a multistep cell-biological process termed the invasion-metastasis cascade, which also is a clear stage of cancer progression that requires the development of angiogenic blood vessels [28]. Cancer cell invasion during angiogenesis is a key process that involves degradation of the basement ECM barriers which allow cells mobility to form new blood vessels. It is the cell-associated MMPs that are responsible for the majority of ECM degradation. The expression of the MMPs is highly regulated since appropriate degradation of matrix would compromise the integrity of tissues [29]. Specifically, MMP-2 along with MMP-9 has a great effect on degrading type IV collagen, which is the most abundant component of the basement membrane. In the present study, we found that, the culture medium of EA.hy926 cells significantly enhanced the invasion ability of the HepG2 cells by improving the release of MMP-2 and MMP-9 in HepG2 cells.

Besides, angiogenesis is essential for tumor growth and metastasis [30]. The tumor associated angiogenesis do not necessarily follow tissue planes, but are effective in supplying oxygen, nutrients and in promoting further growth [31, 32]. To obtain the blood supply, tumor cells can tilt the balance toward stimulatory angiogenic factors to drive vascular growth by attracting and activating cells from the microenvironment of the tumor [33]. In the early phase of tumor development and at the late stages of cancer progression, plenty of molecular pathways and systems have directly or indirectly been implicated in the induction of angiogenesis and in the maintenance of metastasis supporting vascular networks [34, 35]. However, it is noteworthy that the VEGF molecule represents a critical factor that regulates almost all aspects of tumor-induced angiogenesis, like endothelial cell sprouting and assembly, lumen formation [36–38], and also, VEGF expression levels within the tumor environment appear to directly correlate with the overall microvessel density [39]. Another crucial growth factor, EGF, also plays a great role in the process of tumor development [40]. Besides the VEGF and EGF, there are also numerous other cytokines play effective roles in this process. To name a few, Kathleen et.al [41] found that IL-6, CXCL8, or EGF showed upregulation in primary endothelial cells cultured with conditioned culture medium of squamous carcinoma cells through STAT3/Akt/ERK signaling. Ding et.al [42] studied that the expression level of chemokine CXCL9 in CD133+ liver cancer cells was significantly elevated in the culture supernatants of direct co-culture with human umbilical vein endothelial cells by activating the NF-kB.

Accordingly, our results indicated that with the stimulation of the HepG2 cells culture medium, the tube formation of the EA.hy926 cells got markedly improved. Further study showed that both the intracellular and the extracellular expression of VEGF and the receptors of VEGF, Flt-1 and Flk-1 as well as the EGF and EGFR were also increased in these two cell lines after the treatment of exchanging medium. While using ELISA assay to determine whether the VEGF or EGF was released by the cells or just maintained in the exchanging medium, in other words, to ensure the veracity, we designed two time gradient. From the results we can see, as for EA.y926 cells, after treating with HCCM for 30 min, the concentration of VEGF was significantly higher than that of the control, and 60 min later, the concentration was still even notably higher than that of the 30 min. In the aspect of the EGF, we also found that the concentration in both 30 min and 60 min were significantly higher than that of the control. To HepG2 cells, though the concentration of the VEGF under the stimulation of 30 min VECM had no significant difference to that of the control, however, with a longer stimulus time, till 60 min, the concentration was markedly higher than the control and the 30 min, and also, the concentration of EGF after 30 min and 60 min were significantly higher than control. We speculated that there were probably certain amounts of VEGF or EGF in the original medium. Subsequently, the stimulated cells continuously released VEGF and EGF with the exchanging medium, which could increase their concentration. However, it is hard to distinguish the sources of VEGF and EGF from exchanging medium or from stimulated cells by ELISA assay.

A lot of researchers investigated the cross-talk between different cells involved in different signaling pathway. Yu *et al* [43] reviewed the crosstalk associated STAT3-induced signaling pathway between cancer cells and immune cells. Also, the STAT3/Akt/ERK signals contributed to endothelial cells-initiated cross-talk affected on squamous carcinoma cells [41]. In addition, cross-talk between integrins and growth factor receptors are an important signaling mechanism to provide specificity during tumor angiogenesis. Previously studies also demonstrated that the functional activities of growth factors like VEGF and EGF are dynamically and reciprocally controlled by integrin [44]. The fact that growth factor signaling requires the presence of specific integrin subunits and that different cell types express different profiles of integrin may constitute the cellular context determining the outcome of the growth factor signal [45]. However, integrin-induced FAK-Rho GTPases signaling pathway contributed to the cellular crosstalk associated with cell migration is unclear. Integrins are αβ heterodimeric transmembrane proteins implicated in a large number of physiological processes including adhesion to the extracellular matrix, proliferation, survival, migration and differentiation [46]. Each integrin consists of one of 18 α- and one of eight β-subunits, giving rise to a repertoire of 24 different integrin in mammals [47]. It has been believed that integrin and growth factor receptors can interact with one another on multiple levels on the pathway [48]. In traditional models of integrin-growth factor cross-talk, integrin bind to extracellular matrix proteins, and growth factor receptors bind to growth factors [48, 49]. The results suggested that, there was a correlation between the VEGF/EGF and integrin, but we still couldn’t make sure the temporal sequence of the activation.

Integrins are also involved in signal transduction during angiogenesis by stimulating the assembly of intracellular signaling molecules, such as FAK or integrin linked kinase (ILK). Integrin-mediated cell adhesion stimulates the activity of PI3K and its downstream targets of AKT [50]. The domain of integrin cytoplasmic tails is crucial components of signaling pathways involving small GTPases in the regulation of F-actin crosslinking. And the cytoskeleton-associated proteins Paxillin, Vinculin, Talin locate within the focal adhesion complex assemble activated integrin clusters, providing enough adhesive sites to support stable cell attachment by forming FA plaques [51, 52]. FA plaque disassembly drives the migration cycle through activated Rho-family GTPases, which comprises the Rac, Rho and Cdc42, and they are well-studied regulators of cell motility. Rac induces the assembly of focal complexes and actin polymerization during the formation of lamellipodia. Rho induces the formation of stress fibers, whereas Cdc42 induces actin polymerization for the formation of flopodia. In present study, our results showed that the Integrin-FAK-Rho GTPases signaling pathway contributed to enhancing the migration capacity of EA.hy926 cells and the HepG2 cells. These are attributed to activation of Rho-GTPases by antigenic stimuli in exchanging culture medium. In general, the cascade of integrin-FAK-Rho GTPases signaling events in EA.hy926 cells and HepG2 cells showed time-dependent differences and similarities. As for EA.hy926 cells, the expression of integrin subunit α2, α5, Vinculin, RhoA and Rac1 were all increased in the initial 15 sec, while β1. Paxillin and FAK were decreased from 30 min. Similar expression occurred in the HepG2 cells, the level of α2, α5, β1 and β3 advanced in the initial 15 or 30 sec, and together with the αV, the expression of all these integrin subunits in HepG2 cells were reduced from 30 min. Additionally, in consistent with EA.hy926 cells, the expression of Talin was sharply decreased at 5 min and the Paxillin was increased from 15 sec. Also, *t*FAK and *p*FAK expression and the *p*FAK/*t*FAK showed the maximum at 15 min, but gradually decreased from 30 min. As to Rho GTPases, HCCM could significantly upregulated Rac1 at 15sec, RhoA and Cdc42 at 30 sec, respectively, indicating that all of them were increased at initial. It is rather remarkable that a sharp increase in RhoA and Rac1 expression in endothelial cells at 15 sec (Figure 7e), and a similar change in α5 and β1 expression at 30 sec compared to 15 sec in HepG2 cells (Figure 8a). These results further demonstrated that expressions of integrin and Rho GTPases respond to cytokines released from HCCM and VECM in a time-dependent way. We speculated that integrin, as key receptors at cytomembrane, directly contacted with a lot of ligands from exchanging culture medium, experienced a quick response and showed a sharp increase at transient duration (15 and 30 sec). Also, the RhoA and Rac1, as the important members of Rho GTPases family, result in actin assembly by regulating lamellipodia and filopodia at leading end of cells, which could be perceived with external signaling, and regulated by integrin simultaneously. This study explored the interaction of vascular endothelial cells and hepatocellular carcinoma cell associated with integrin-FAK-Rho GTPases signaling pathway. Importantly, these findings suggest that antiangiogenic therapies targeted at the blockade of this pathway may decrease the rate of local recurrence and minimize the morbidity associated with field cancerization.

## MATERIALS AND METHODS

Human vascular endothelial cells lines, EA.hy926 cells (Hematology Research Instituteof Jiangsu Province, China) instead of primary vascular ECs were used in this study.

It is a fusion cell line made by human umbilical vascular endothelial cells (HUVECs) and the human lung carcinoma epithelial cell A549. It retains the most characteristics of HUVECs, including the expression of endothelial adhesion molecules and human factor VIII-related antigen. Using this cell line, Varga *et.al* [53] previously studied the inhibition efficiency of a specific compound *in vitro* angiogenesis by interfering with endothelial cell migration and tube formation processes.

Hepatocellular carcinoma (HCC) is one of the commonest malignant diseases in the world. HepG2 cells (Hematology Research Institute of Jiangsu Province, China) are adherent, epithelial-like cells growing with small aggregates. These cells secrete a variety of major plasma proteins like albumin, transferrin and the acute phase proteins fibrinogen, α2-macroglobulin, α1-antitrypsin and plasminogen, etc. Therefore, HepG2 cells were used as a representative cancer cell line for present study.

### Cell culture and reagents

Both EA.hy926 cells and HepG2 cells were maintained in RPMI-1640 complete growth medium (Invitrogen Company, USA), supplemented with 10% fetal bovine serum (FBS, Gibco BRL, USA), 2 mM L-Glutamine, 100 U/mL penicillin, 20 mmol/L HEPES (Sigma, USA), 2‰ NaHCO_3_ and 50 mg/mL streptomycin (Beyotime Institute of Biotechnology). Besides, 2% HAT (containing hypoxanthine, aminopterin and thymidin, Sigma, USA) were added to the medium used for EA.hy926 cells. Both of EA.hy926 and HepG2 cells were cultured in an incubator with 5% CO_2_ at 37°C.

To build an interaction system, we exchanged the culture conditioned medium of these two cell lines. That is to say, HepG2 cells and EA.hy926 cells with 90% confluence were starved in RPMI-1640 culture medium with serum-free overnight, and then the culture medium was collected and centrifuged to separate the suspended cells from the medium. Subsequently, the EA.hy926 cells were cultured with collected hepatocellular carcinoma HepG2 cell culture medium (HCCM), and HepG2 cells were cultured with vascular endothelial EA.hy926 cells medium (VECM), respectively.

### MTT assay

MTT (3-(4, 5-cimethylthiazol-2-yl)-2, 5-diphenyl tetrazolium bromide) assay is a test to check metabolic activity of proliferating cells *in vitro* conditions. HepG2 cells and EA.hy926 cells were seeded in 96 well plates respectively, and incubated overnight, and then the culture medium was replaced with the exchanging medium. After 24 hours of incubation, medium was removed, and 20 μL MTT (5 mg/mL) was added in each well and plates were kept for 4 hours in incubator. Then 150 μL of dimethyl sulfoxide (DMSO) were added and shacked gently for 10 min at room temperature in a shaker. The intensity of the color developed was absorbed at 490 nm in a multimode microplate reader (Bio-Rad, USA). Each experiment was performed in triplicates and the same protocol was followed until the completion of the experiment.

### Angiogenesis assay

The tube formation was assayed by *in vitro* Matrigel angiogenesis assay. Briefly, HepG2 cells and EA.hy926 cells were seeded onto Matrigel-coated wells of a 96-well plate and cultured in serum-free RPMI1640 medium, RPMI1640 medium with IL-8, and the exchanging medium for 4h in 37°C and 5% CO_2_. Triple wells were set for each group, and the formation of the tube was examined by a phase-contrast microscopy (Olympus, CK2), and the number of the network structure was quantified by randomly selecting 5 fields per well.

### Cell migration——scratch-wound assays

Scratch-wound assay was used to measure and evaluate migration ability of EA.hy926 cells and HepG2 cells under the condition of stimulation with exchanging culture medium. EA.hy926 cells and HepG2 cells were cultured on the 6-well plates until monolayer confluence. Then the cells were starved and synchronized at serum-free conditions overnight. A uniform scratch (about 500 μm width) was performed in the cell monolayer using a sterile micropipette tip. After washing the slides gently with PBS three times, the exchanging medium was added into the plate. Then the cells were cultured in an incubator containing 5% CO_2_ at 37°C. Three images of the wounds were randomly chosen at 0 h and photographed consecutively at 0, 4, 12 and 24h under static culture using an inverted microscope (CK2, Olympus, Japan). The cell migration distance at the end of each recording period was calculated as the difference between the end length and the original length of the wounded edge.

### Cell invasion——transwell assay

1×10^5^ HepG2 cells deprived of serum overnight were seeded over 8 μm polycarbonate filters of the upper compartment of Transwell chambers in RPMI1640 without serum. Different conditions were tested comparing the effects of serum free RPMI1640 alone (control) and the exchanging medium added to the lower chamber for 48 h at 37°C. Then the cells were washed with PBS, and fixed with 4% paraformaldehyde for 15 min; after that, the cells were stained with 1% crystal violet for 10 min, washed by PBS and counted in 5 random fields under an inverted microscope (CK2, Olympus, Japan). Triple wells were set for each group, and this experiment was performed in triplicates.

### F-actin staining

After culturing EA.hy926 cells and HepG2 cells in the 24-well plates with 70-80% confluence, cells were washed with PBS and incubated with the exchanging medium for 60 min. Then they were fixed with 4% paraformaldehyde for 10 min and were incubated with BODIPY (1:100, Invitrogen™, USA) for 30 min, then DAPI (4’, 6’-diamidino-2-phenylindole) with 1:800 dilution was added and co-incubated for 30 min at 37°C. Samples were observed by laser scanning confocal microscopy (Leica TCS SP5, Germany).

### ELISA (enzyme linked immunosorbent assay)

To examine the release of VEGF and EGF in respective endothelial cell and hepatocellular carcinoma cells under the conditioned medium, ELISA was performed. HepG2 cells and EA.hy926 cells were seeded in 24-well plates and incubated overnight. After discarding the medium and washing the cells with PBS, incubated the cells in the exchanging medium for 0, 30, and 60 min, then the medium was centrifuged at 1000 rpm for 10 min, and collected the supernatant which is the antigen needed in this assay. Polystyrene microtiter plate wells were coated with the antigen (50 μL per well, diluted in coating buffer) at 4°C overnight. After washing once with PBST (PBS containing 0.05 % Tween 20), the plates were blocked with skimmed milk overnight. Then incubated with diluted (1:100) primary antibodies including VEGF and EGF at 37°C for 1h, after washing four times with PBST, the plates were incubated with 100 μL diluted horseradish peroxidase-conjugated goatanti-mouse antibody or goat anti-rabbit antibody for incubation for 1h at 37°C. Then washed with PBST and TMB (3, 3’, 5, 5’-Tetramethylbenzidine) was added to each well. The reaction was stopped by the addition of H_2_SO_4_. The optical density was measured 450 nm using spectrophotometer. All individual samples were assayed induplicate.

### Western blotting assay

Cells cultured for 48 h with 60-80% confluence were treated with the exchanging medium for 0, 15, 30s, and 5, 15, 30, 60 min and then washed three times with PBS before disintegrated by 50 μL cell lysis solution. The total protein was collected and centrifuged with 14,000 rpm at 4°C for 10 min, quantified by enhanced bicinchoninic acid (BCA) assay kit (Beyotime Biotechnology Co., LTD, Beijing, China). Equal amounts of protein (30 μg) were loaded onto each lane of a 10% SDS-PAGE gel. After gel electrophoresis and membrane transferring, polyvinylidene difluoride membranes (PVDF, GE Healthcare) were blocked for 2h in 5% BSA in TBST buffer (20 mM Tris-HCl [pH 8.0], 150 mM NaCl, 0.05% Tween 20) at 37°C. Membranes were incubated with primary antibodies including VEGF, Flt-1, Flk-1, EGF, EGFR, MMP-2, and MMP-9, as well as FAK, integrins, Talin, Paxillin, Vinculin, RhoA, Rac1 and Cdc42 (Santa Cruz, Inc., USA) overnight at 4°C. HRP binding secondary antibodies were incubated for 2h at 37°C. Bands were visualized by enhanced chemiluminescence and Molecular Image^®^ChemiDoc™ XRS^+^ system with Image Lab™ Software. The tests were performed three times and quantification was done and analyzed by Image J 1.44p software (National Institutes of Health, USA). The intrinsic controls (β-actin) were used to guarantee the uniformity of equal loaded protein among all groups, and the values of control have been normalized as “1” for consistency to compare.

### Immunofluorescence staining

After culturing for 48h, cells were washed with PBS and incubated with the exchanging medium for 0, 30, 60 min, and then fixed with 4% paraformaldehyde and permeabilized with 0.3% Triton X-100 for 10min. Following that, samples were blocked by adding 1% BSA (bovine serum albumin, w/v) for 15 min. Cells were incubated in the primary antibody solution (anti-VEGF and anti-Flk-1, anti-EGF and anti-EGFR, respectively, 1:100 dilution) at 4°C overnight. The secondary FTIC-conjugated immunoglobulin (Goat anti-mouse IgG, Biosynthesis biotechnology Co., LTD, Beijing, China) and TRITC-conjugated immunoglobulin (Goat anti-rabbit IgG, Biosynthesis biotechnology Co., LTD, Beijing, China) were mixed and incubated at 37°C for 60 min to conjugate respective primary antibody. The DAPI with 1:800 dilution was added for nuclei staining for 40 min. Samples were observed by laser scanning confocal microscopy (Leica TCS SP5, Germany).

### Statistical analysis

The data obtained in this study were analyzed using statistical software SPSS 11.5 (SPSS, Inc., Chicago, Illinois) and reported as means±standard deviation. Data obtained from different treatment groups were statistically compared. To reveal differences among the groups, one-way ANOVA followed by Tukey’s test was used. Differences were considered significant at *P*< 0.05.

## SUPPLEMENTARY MATERIALS FIGURE



## Abbreviations

BSA: bovine serum albumin
DMSO: dimethyl sulfoxide
ECs: endothelial cells
ECM: extracellular matrix
EGF: epidermal growth factor
EGFR: epidermal growth factorreceptor
FA: focal adhesion
FAK: focal adhesion kinase
HCC: hepatocellular carcinoma
HCCM: hepatocellular carcinoma HepG2 cell culture medium
ILK: integrin linked kinase
MMPs: matrix metalloproteinases
*p*FAK: phosphorylated FAK
PVDF: polyvinylidenedifluoridemembranes
*t*FAK: total FAK
VECs: vascular endothelial cells
VECM: vascular endothelial EA.hy926 cells medium
VEGF: vascular endothelial growth factor
