# Supplementary material for: Cross-talk mechanism between endothelial cells and hepatocellular carcinoma cells via growth factors and integrin pathway promotes tumor angiogenesis and cell migration
Source: Oncotarget. 2017 Jun 27;8(41):69577–93. doi: 10.18632/oncotarget.18632 (PMC5642501; doi:10.18632/oncotarget.18632)
Supplement: Supplementary file 1 [file oncotarget-08-69577-s001.pdf]

Cross-talk mechanism between endothelial cells and hepatocellular carcinoma cells via growth factors and integrin pathway promotes tumor angiogenesis and cell migration

SUPPLEMENTARY MATERIALS

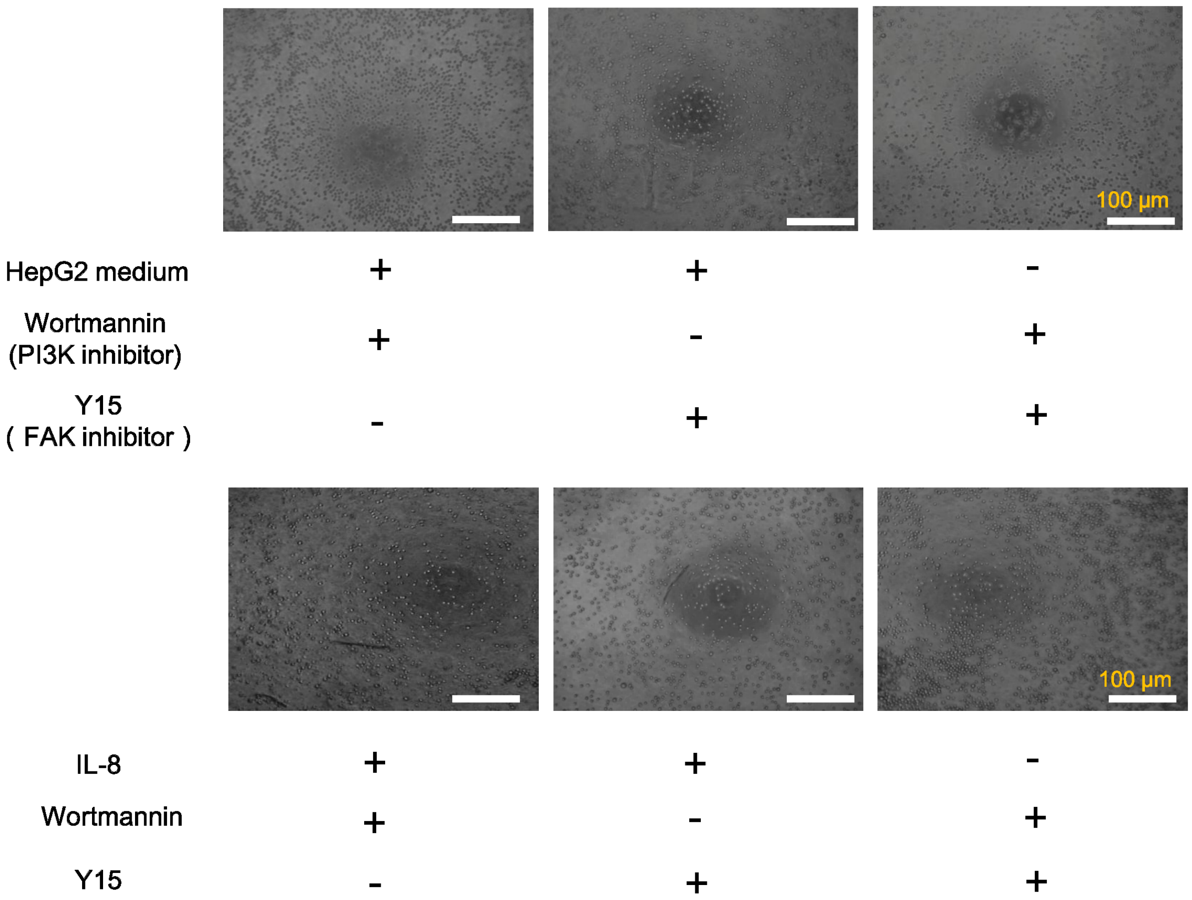

Supplementary Figure 1: PI3K and FAK inhibitor blocked the tube formation of EA.hy926 cells.
